# *Frankia alni* Carbonic Anhydrase Regulates Cytoplasmic pH of Nitrogen-Fixing Vesicles

**DOI:** 10.3390/ijms24119162

**Published:** 2023-05-23

**Authors:** Petar Pujic, Lorena Carro, Pascale Fournier, Jean Armengaud, Guylaine Miotello, Nathalie Dumont, Caroline Bourgeois, Xavier Saupin, Patrick Jame, Gabriela Vuletin Selak, Nicole Alloisio, Philippe Normand

**Affiliations:** 1 Ecologie Microbienne, Centre National de la Recherche Scientifique UMR 5557, Université de Lyon, Université Claude Bernard Lyon I, INRAE, UMRA1418, Cedex, 69622 Villeurbanne, France; lcg@usal.es (L.C.); pascale.fournier@univ-lyon1.fr (P.F.); nicole.alloisio@orange.fr (N.A.); 2 Departamento de Microbiología y Genética, Facultad de CC Agrarias y Ambientales, Universidad de Salamanca, Plaza Doctores de la Reina, 37007 Salamanca, Spain; 3 Département Médicaments et Technologies pour la Santé (DMTS), SPI, Université Paris Saclay, CEA, INRAE, 30200 Bagnols-sur-Cèze, France; jean.armengaud@cea.fr (J.A.); guylaine.miotello@cea.fr (G.M.); 4 Laboratoire DEEP, INSA, Cedex, 69622 Villeurbanne, France; nathalie.dumont@insa-lyon.fr; 5 Institut des Sciences Analytiques, UMR 5280, Université de Lyon, CNRS, Université Claude Bernard Lyon 1, 5 rue de la Doua, 69100 Villeurbanne, France; caroline.bourgeois@isa-lyon.fr (C.B.); xavier.saupin@isa-lyon.fr (X.S.); patrick.jame@isa-lyon.fr (P.J.); 6 Institute for Adriatic Crops and Karst Reclamation, Put Duilova 11, 21000 Split, Croatia; gabriela.vuletin.selak@krs.hr; 7 Centre of Excellence for Biodiversity and Molecular Plant Breeding (CoE CroP-BioDiv), Svetošimunska Cesta 25, 10000 Zagreb, Croatia

**Keywords:** homeostasis, acidity, symbiosis

## Abstract

A phyloprofile of *Frankia* genomes was carried out to identify those genes present in symbiotic strains of clusters 1, 1c, 2 and 3 and absent in non-infective strains of cluster 4. At a threshold of 50% AA identity, 108 genes were retrieved. Among these were known symbiosis-associated genes such as *nif* (nitrogenase), and genes which are not know as symbiosis-associated genes such as *can* (carbonic anhydrase, CAN). The role of CAN, which supplies carbonate ions necessary for carboxylases and acidifies the cytoplasm, was thus analyzed by staining cells with pH-responsive dyes; assaying for CO_2_ levels in N-fixing propionate-fed cells (that require a propionate-CoA carboxylase to yield succinate-CoA), fumarate-fed cells and N-replete propionate-fed cells; conducting proteomics on N-fixing fumarate and propionate-fed cells and direct measurement of organic acids in nodules and in roots. The interiors of both in vitro and nodular vesicles were found to be at a lower pH than that of hyphae. CO_2_ levels in N_2_-fixing propionate-fed cultures were lower than in N-replete ones. Proteomics of propionate-fed cells showed carbamoyl-phosphate synthase (CPS) as the most overabundant enzyme relative to fumarate-fed cells. CPS combines carbonate and ammonium in the first step of the citrulline pathway, something which would help manage acidity and NH_4_^+^. Nodules were found to have sizeable amounts of pyruvate and acetate in addition to TCA intermediates. This points to CAN reducing the vesicles’ pH to prevent the escape of NH_3_ and to control ammonium assimilation by GS and GOGAT, two enzymes that work in different ways in vesicles and hyphae. Genes with related functions (carboxylases, biotin operon and citrulline-aspartate ligase) appear to have undergone decay in non-symbiotic lineages.

## 1. Introduction

Actinobacterial genus *Frankia* contains several symbiotic lineages that fix nitrogen within the root nodules of plants belonging to the *Rosales*, *Fagales* and *Cucurbitales* orders [1]. A phylogenetic analysis showed that these symbiotic *Frankia* formed three clusters [2]. Conversely there are numerous strains that have lost various symbiotic features; these form a coherent cluster [3]. These non-symbiotic strains fall into two categories: those that are non-infective such as CN3 or DC12 [4,5] and those strains that are infective but non-nitrogen-fixing such as EuI1c or AgB1.9 [6,7]. The relative position of the clusters has been found to fluctuate depending on the marker used or the strains studied, [8,9] but all are robust.

It has been estimated that the symbiotic *Frankia* strains diverged from other soil actinobacteria around 80–100 MY [2], a time length that corresponds roughly to that of the emergence of the plant lineages in symbiosis with *Frankia,* predating the emergence of the Fabaceae which is estimated at 60 MY [10]. This is thus a lengthy period of time that could have impacted the *Frankia* genomes with genes which are non-essential for symbiosis, presumably costly to express and dispensable in free-living lineages, and during which these genes could have been subject to negative evolutionary pressure leading them to be lost in a manner similar to that occurring in non-symbiotic host plant lineages in relation to symbiosis-associated genes [1].

More than 40 *Frankia* genomes from all clusters have become available since the first ones were published in 2007 [11,12]. These genomes have been compared and have been found to fluctuate in terms of parameters such as size [11], GC% [12], codon usage [13] and Ka/Ks [14] as they have been subjected to different evolutionary pressures in the soil, on the one hand, or in plant tissues, on the other. There has been massive genome erosion between non-sporulating (Sp−) and sporulating strains (Sp+) [15], for instance, in *Alnus*-infective strains that have lost around 2500 genes, or one third of the total number.

There have also been studies targeted at groups of genes which have shown the presence of canonical *nod* genes in some cluster 2 strains [16,17] and one cluster 3 strain [18], the absence of *nif*, *sodF* and *rhb* genes in non-symbiotic cluster 4 strains [12], the presence of *celA-bcsA* genes in symbiotic strains [19] and the loss of saprophytic genes such as *gvp* in cluster 1c strains and cluster 2 strains that have reduced genomes [11,16]. These genomes are thus a wealth of data that can be analyzed to try to understand the genetic determinants underpinning symbiosis in the absence of a proven genetic transformation protocol. We decided to compare symbiotic and non-symbiotic genomes to determine if the estimated 100 MY since their separation had resulted in the loss of symbiosis-associated determinants in those strains that had lost the ability to establish nodules. One such determinant that emerged was the *can* genes that code for carbonic anhydrase (CAN), an enzyme that converts CO_2_ into carbonic ions and protons mainly for pH homeostasis and the functioning of carboxylases. There are 23 of these genes in *Frankia alni*, and we investigated them from a physiological viewpoint.

## 2. Results

### 2.1. Genome Mining

When a search was made for those proteins conserved at a threshold of 50% identity between symbiotic cluster1 (cl1) strains (ACN14a, QA3, CcI3), cluster 2 (cl2) strains (BMG5.1 and Dg1) and cluster 3 (cl3) strains (EaN1pec and EUN1f) and absent from non-symbiotic non-infective cluster 4 (cl4) strains (CN3 and DC12), 108 hits were obtained (Table 1). Among those genes retrieved were nif (nitrogenase, FRAAL6800-6814); rhbBCEF (rhizobactin-related metabolite; FRAAL6422- FRAAL6426); sodF (Fe-superoxide dismutase, FRAAL4337); accA (acetyl carboxylase, FRAAL3196); argF (argininosuccinate lyase, FRAAL5202) and two can (carbonic anhydrase; FRAAL1222, FRAAL4889). These two CANs belong to the Beta class prevalent in prokaryotes which is more active than the Gamma CANs [20]. The can genes were found to be present in some genomes belonging to cluster 4, such as Frankia AgW1.1 and AgB1.8 strains, isolated from *Alnus* and able to reinfect *Alnus* but unable to fix nitrogen in pure culture or in vitro.

When a search for gene annotations was carried out in the MAGE data base using “carbonic anhydrase” as keyword, supplementary hits were obtained in several lineages including *Frankia alni*, FRAAL2078, that would code for a distant Gamma CAN with homologs in all Frankia strains. Finally, the same keyword search yielded a Beta CAN present in cluster3 and cluster4 strains with a very low ID (<30% AA) with FRAAL1222 and FRAAL4889 CANs.

### 2.2. Cells Growth

**Figure 1:**
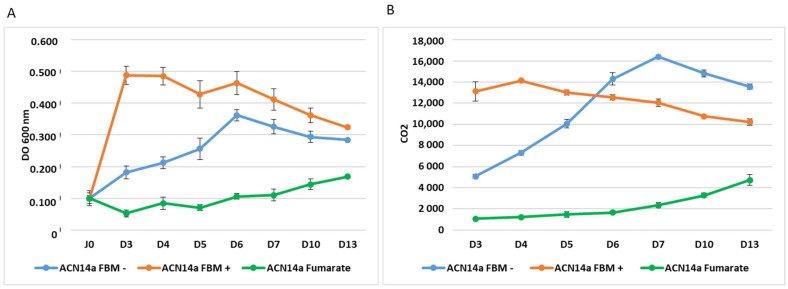
(**A**) Growth of *Frankia alni* strain ACN14a (DO 600 nm) in FBM medium with 5 mM propionate and 5 mM NH_4_Cl (yellow dots), in FBM medium with 5 mM propionate without NH_4_Cl (blue dots) and in FBM medium without propionate but with 5 mM fumarate and without NH_4_Cl (green dots) followed over a time course of 13 days; (**B**) CO_2_ in the gas phase over strain ACN14a (in the same conditions as in (**A**)). Bars are +/− standard error.

Growth, N-fixation and CO_2_ levels were monitored over liquid N-fixing and N-replete cultures. Frankia grew rapidly on propionate and ammonium, reaching a plateau after only 3 days, whereas it took 6 days when fixing nitrogen on propionate, and on fumarate it took 13 days (Figure 1). The CO_2_ in the propionate+ammonium-fed culture reached a plateau at day 4, whereas in the propionate-fed nitrogen fixing culture, CO_2_ increased steadily over seven days as the cells multiplied. CO_2_ in the fumarate-fed culture was low at first and increased slowly thereafter.

### 2.3. Physiology Measurements of Cells

**Figure 2:**
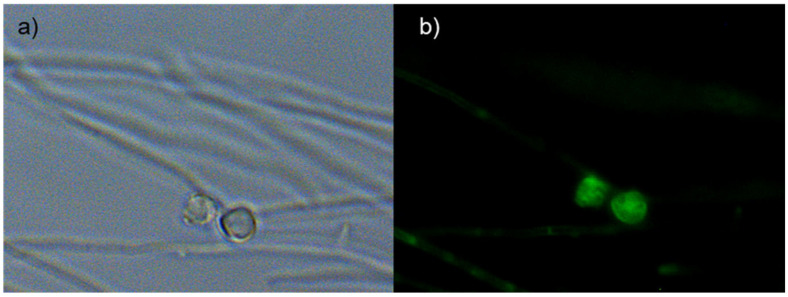
Free cells cultures of *Frankia alni* ACN14a grown for 7 days in BAP^−^ medium observed under visible light and UV microscope. Phase contrast (**a**) and green filter DND-189 probe-treated (**b**). Strong fluorescence indicative of acidity is found inside N_2_-fixing *Frankia* vesicles.

**Figure 3:**
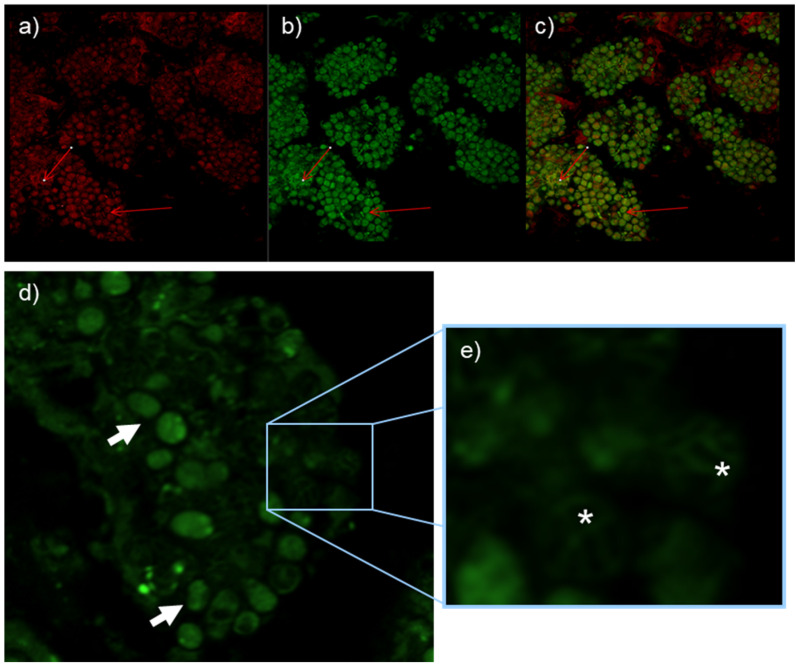
*Alnus glutinosa* nodules resulting from inoculation of *Frankia alni* ACN14a. Nodules treated with DND-99 and SYTO-9 under red filter (**a**) green filter (**b**) and overlay (**c**) show acidity inside vesicles. Nodules treated with DND-189 probe show absence of acidity in intervesicular spaces (arrows), (**d**) and in intermembrane spacers (*), (**e**).

*Frankia alni* cells grown on an FBM-medium were stained with DND-189 and DND-99 fluorescent probes and observed under fluorescent light with filters as well as with SYTO-9, which showed the vesicles to be fluorescent (Figure 2) thus indicating their pH is acidic, more so than that of the hyphae that did not fluoresce with the pH-responsive stains. After staining 21 days post infection (dpi), nodule sections showed strong fluorescence with DND-99 and SYTO-9 stains, thus indicating acidity was found inside the cytoplasm of the vesicles (Figure 3).

### 2.4. Gene Expression

**Figure 4:**
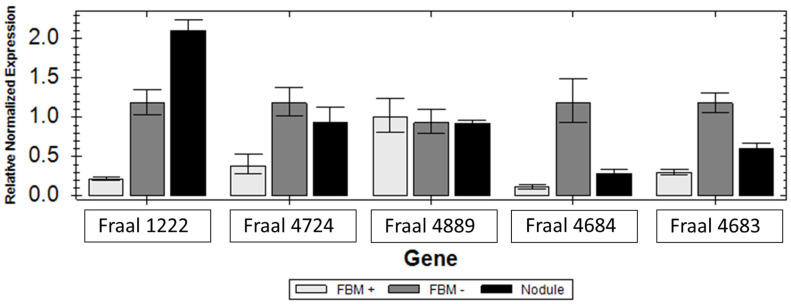
Relative expression of *can1* (FRAAL1222), *can2* (FRAAL4889), *ctp* (FRAAL4724), *cpsA* and *cpsB* (FRAAL4684 and FRAAL4683) genes in *Frankia alni* ACN14a grown under nitrogen-free conditions in FBM− (grey) media, with 5 mM ammonium in FBM+ media (light grey) and in symbiosis within *Alnus glutinosa* nodules (dark).

Expression levels of *can1* (FRAAL1222), *can2* (FRAAL4889), *ctp* (FRAAL4724) and two carbamoyl phosphate synthases *cpsA* (FRAAL4684) and *cspB* (FRAAL4683) are shown in Figure 4. The *can1* and *ctp* genes were highly upregulated under nitrogen starvation in FBM− media and in symbiosis. The second *can* gene (FRAAL4889) was not differentially expressed under all conditions tested. The two carbamoylphosphate synthase genes, *cpsA* and *cpsB* genes, were up-regulated in free-living cultures without nitrogen and down-regulated in symbiosis. A low level of expression was observed under growth as free culture in FBM+ with supplied ammonium.

### 2.5. Proteomics

The proteomic analysis results of fumarate- and propionate-fed cells under nitrogen fixing conditions are shown in Table 2 with the 40 most up-regulated proteins of each condition. Fumarate-fed cells had a high overabundance of acyl-CoA synthase, three dicarboxylate transporters and proteases. Propionate-fed cells had carbamoyl-phosphate synthase, nitrogenase, SHC and lipid synthase as the most overabundant proteins.

### 2.6. Metabolomics of Organic Acids

The small organic acids present in roots and 21 dpi nodules were formic, acetic, pyruvic, propionic, malonic, malic, fumaric, α-ketoglutaric, succinic, oxalic and citric acids (Table 3). Among these, citric, malic and oxalic acids were the most abundant, while α-ketoglutaric acid fumaric acid and the monocarboxylates pyruvic acid and acetic acid were those with the highest FCs of 2.1, 2.1, 1.9 and 1.6, respectively, in nodules relative to roots.

## 3. Discussion

*Frankia* symbiotic determinants are still poorly understood mostly due to the absence of an effective genetic transformation protocol. Omics, transcriptomics and proteomics are promising new approaches for the study of plant—bacteria interactions, and have been used to circumvent this drawback in *Frankia*. A transcriptomic study of 21 dpi symbiotic cells showed upregulation of the genes *nif*, *hup*, *suf* and *shc* [21]. More recently, it was shown that cellulase and cellulose synthase were upregulated upon early (2 dpi) contact even although *Frankia alni* feeds neither on glucose nor on cellulose [19], suggesting a role in penetration into the root hair. However, few hints have emerged through omics on the nature of the trophic relations between symbiont and host.

Carbonic anhydrase (CAN) is a zinc-dependent metallo enzyme that catalyzes the reversible reaction of CO_2_ with water into carbonic ions and protons, thereby modifying pH and permitting the subsequent action of carboxylases. In animal lungs, carbonic anhydrase converts blood bicarbonate to carbon dioxide that is then exhaled. In the stomach, the CAN of parietal cells produces acid, in the kidneys it helps maintain the blood pH level by forming protons that are then secreted into urine. In plants, CAN helps increase chloroplast CO_2_ concentration to facilitate the carboxylation of RuBisCO. Bacteria need to adapt to niches with low pH, for example *Helicobacter pylori* in the stomach [22] or *Mycobacterium tuberculosis* in macrophages [23], and CAN has been shown to permit adaptation to these biotopes. An *Escherichia coli can-* mutant can only survive if the atmospheric partial pressure of CO_2_ is high or during anaerobic growth in a closed vessel at low pH where copious CO_2_ is generated endogenously [24]. Pathogen CANs are also targets of specific enzyme inhibitors and fight antibiotic resistant cells. This mechanism is known to be used against *Mycobacterium tuberculosis* [23,25], *M. leprae* [26], *Legionella pneumophila* [22] and several other bacteria [27]. Thus, CAN activity is a critical feature for these bacteria. Many CAN enzymes have been described with less sequence identity which suggests the convergent evolution of the enzymatic function [28]. Carboxylases play important roles in the cell; for instance phosphoenolpyruvate carboxylase feeds pyruvate into the TCA, propionyl-CoA carboxylase yields methylmalonyl-CoA for feeding succinyl-CoA into the TCA, acetyl-CoA carboxylase yields malonyl-CoA for the lipid synthesis or carbamoyl phosphate synthetase that then permit L-ornithine to transform into L-citrulline.

Propionate and acetate are known antibacterial compounds which are active against a wide range of microbes. Their mechanism of action has been postulated to be a drop in cytoplasmic pH [29] or synergy and potentiation of the anti-bacterial activity of transition metals [30]. They can freely cross membranes when they are not charged (they are neutral at their pKa, 4.8 for acetate, 4.9 for propionate, and much lower at 2.4 for pyruvate) and thus require no dedicated transporter [31] except at low concentrations or at a pH above neutrality [32]. They are used as food preservatives, yet propionate and acetate are the most efficiently used carbon sources in *Frankia* [33], presumably because *Frankia* has been selected over millions of years to use them and can counter their toxic properties. One study claimed that one *Frankia* strain needed a transporter for propionate, but it is probable that the inhibitors used inhibited incorporation into the TCA and the labeled propionate could then simply move freely in and out of the cells [34]. The present study found three expressed dicarboxylate transporters when *Frankia* was fed the dicarboxylate fumarate but no likely propionate transporter when fed propionate. Furthermore, the onset of nitrogen fixation was much more rapid when *Frankia* was fed monocarboxylates than with dicarboxylates, suggesting there was no need for the synthesis of a dedicated monocarboxylates transporter [33].

Symbiotic *Frankia* lives in a sheltered niche, the root nodular cortex, but it nevertheless faces strong physiological constraints. One of those constraints is pH homeostasis as it is modified by several enzymes such as upregulated nitrogenase that requires protons and ATP to reduce dinitrogen to ammonium or to uptake hydrogenase that recycles hydrogen into protons and ATP. *Rhizobium,* that has similar constraints, has its peribacteroid space made acidic by the host to provide a proton-motive force, presumably to permit uptake of malate and activate proteases [35] and also to trap fixed nitrogen as ammonium and not as gaseous ammonia that would otherwise escape at a pH above 6 [36].

Among *Frankia* cells, the hyphae and vesicles differ from each other in terms of physiology, with hyphae having a complete ammonia assimilation pathway (GS and GOGAT) and vesicles only having an active GS that would lead to large amounts of glutamine under low O_2_ [37]. It has been hypothesized that glutamine could then diffuse toward the plant as a consequence of several peptides which are upregulated in nodules and which increase porosity towards AA [38]. One of the vesicles’ main characteristics is to have a multi-lamellate wall with hopanoid lipids providing a passive diffusion barrier to oxygen, yielding an anaerobic environment in which nitrogenase can function [39]. GSII, the primary enzyme responsible for NH_4_^+^ assimilation, was found to have an optimum pH of 6.4 [40], whereas GOGAT and GDH had optimums of 7.6 and 8.6, respectively [37], which is 1 or 2 pH units above that of GSII. This difference in the pH response of the two enzymes could be explained by the more acidic conditions in the vesicles and is consistent with the vesicles only having GSII activity while the hyphae have a complete cycle. In this scenario, the GS would be complemented by carbamoyl-phosphate synthase (CPS) and ornithine carbamoyl transferase (OCT) which use HCO_3_^−^ to incorporate NH_4_^+^ into ornithine and yield citrulline [41]. OCT is also found in the phyloprofile, with only a distant homolog (32% identities) present in non-symbiotic lineages, with its closest relatives being found in the *Planctomyces*.

Propionate is synthesized through the catabolism of chlorophyll, valine and fatty acids [42]. The synthesis of acetate as acetyl-CoA in plant cells occurs through pyruvate dehydrogenase entering the TCA cycle and yields lipids and flavonoids [43]. Propionate is known to be upregulated as a response to different situations such as drought [44] and high levels of CO_2_. The catabolism of lipids is also known to yield acetate [43]. However, in nodules we see much higher levels of dicarboxylates than of monocarboxylates, suggesting *Frankia* could be fed a mixture of both types of organic acids, with monocarboxylates also serving to modulate cytoplasmic acidity.

Nodules are known to fix CO_2_ at five times the level of adjacent roots [45], with the labeled carbon ending up as amino acids (glutamate, aspartate and citrulline) and organic acids (malate, citrate and succinate) through the action of PEP carboxylase or pyruvate carboxylase, an enzyme that would be active when the carbon source is pyruvate. *Frankia* strain ArI3 in pure culture was also shown to fix more nitrogen when supplied supplementary CO_2_, but only when fed monocarboxylates [46] and not TCA intermediates such as succinate, malate, etc. Pyruvate is assimilated through a dedicated carboxylase and transformed into oxalacetate; propionate is assimilated through the propionyl CoA carboxylase and transformed into succinate. Both oxalacetate and succinate use biotin, and acetate is assimilated through acetyl-CoA carboxylase into malonyl-CoA and lipids. Another possible pathway for the assimilation of propionate is the methyl citrate cycle that is also present in symbiotic strains and absent in non-symbiotic strains. This pathway does not require carboxylase and could function to complement the methyl-malonyl pathway. It has long been assumed that the host feeds *Frankia* with dicarboxylates since the expression of *AgDCAT*, a transporter specific for dicarboxylates, was nodule-specific [47]. However, monocarboxylates could also contribute to the nutrition of *Frankia* since pyruvate, for instance, is present in the roots and nodules of *Casuarina* and *Alnus* [48]. The transport of monocarboxylates appears to be facilitated in *Coryebacterium* by a specific sodium solute symporter at a high pH, but import can nevertheless occur through simple diffusion at a neutral pH [32]. When *Frankia* is fed a dicarboxylate, it upregulates three transporters, all of which were not up-regulated in 21 dpi nodules [21].

The most overabundant proteins in propionate-fed in-vitro-grown cells were CPS subunit, proteins that use ammonium, phosphate and HCO_3_^−^ to yield carbamoyl that could then be used to transform L-ornithine into L-citrulline, the compound shown to carry the most labels after ^15^N has been fed to *A. glutinosa* [49]. This could be a pathway for the assimilation of ammonium which is complementary to the GS-GOGAT pathway. *Frankia* has been shown to be able to use L-ornithine as source of nitrogen without inhibition of nitrogenase. This could also serve to select efficient nitrogen fixers that can provide NH_4_^+^ to quench carbonate and maintain a slightly acidic pH.

The genes encoding CANs are differentially expressed in nodules (FRAAL1222: 1.20; FRAAL2078: 0.86; FRAAL4724: 1.15; FRAAL4889: 0.71, relative to BAP^+^, [21]), a process which is compatible with the requirement for CO_2_ when grown on propionate in vitro and the very high efficiency of the CAN that have a low Km [50]. It is also compatible with the host feeding the symbiont a mixture of dicarboxylates and monocarboxylates. The amount of organic acids measured in this study and by previous authors [33,48] do lead to a certain conclusion in relation to the photosynthates fed to the microsymbiont because of likely compartmentation in the mitochondria, chloroplasts and vacuoles. Nevertheless yet these organic acids are good candidates for the role.

Carbonic anhydrase would thus be added to the list of symbiosis-related genes such as those coding for nitrogenase, hopanoid biosynthesis, iron-sulfur cluster synthesis, hydrogen-uptake [21] and cellulase and cellulose synthase [19]. Similar to hydrogen-uptake and cellulase, there are copies that have diverged early in the evolutionary history of the genus. Whenever genetic editing becomes practical, it will be of interest to assay this symbiosis determinant.

## 4. Materials and Methods

### 4.1. Genome Mining

Genome comparisons were made on the Mage platform [51]. Conserved genes present in representative genomes from clusters 1, 2 and 3 were extracted, and those present in non-symbiotic lineage 4 were subtracted using as the threshold 50% of identities over 80% of the length of the shorter AA sequence. For cluster 4, only CN3 [52] and DC12 [5] were used because these are unable to induce nodules in any of the hosts tested other than EuI1c and AgB1.9 that can induce inefficient nodules [53]. A list of the strains and a flow chart of research directions are given in the Supplementary Materials (see Supplementary Materials Table S1 and Figure S1).

### 4.2. Plant Growth

*Alnus* seeds were surface-sterilized and germinated as described previously [19]. Seedlings were transferred to opaque pots, grown with N-free medium and inoculated with *Frankia alni* as before [1].

### 4.3. Culture of Cells and Physiology Measurements of Cells

Growth, N-fixation and CO_2_ levels were followed in FBM medium [54], both with and without 5 mM ammonium, and in the same medium with 5 mM fumarate instead of propionate. Growth was monitored as previously [33] and CO_2_ in the headspace was measured in the flask every day using a gas chromatograph (Micro GC R3000, SRA Instrument, Marcy L’Etoile, France) as described previously [55].

### 4.4. Microscopy

The staining of nodule slices and FBM− grown *Frankia* was carried out with DND-99 probes (LysoTracker Red) or DND-189 (LysoSensor Green, Molecular Probes, Eugene, OR, USA) in a 50 mm Tris pH = 7 buffer. Excess dye was removed by a short wash with buffer and samples were observed using a confocal microscope (LSM510 META, Carl Zeiss, Le Pecq, France) using a plan-Apo 63×/1.2 NA water immersion objective.

### 4.5. Phylogenomics

The amino acid sequences of the carbonic anhydrase genes from the representative Frankia species and related actinobacteria were retrieved using the BlastP method [26]; and were aligned using the Seaview platform [56] implementing the ClustalW2 v2.1 software [57]. The alignment was then used for a Maximum Likelihood tree reconstruction approach [58] with a bootstrap of 1000 replicates [59] to assess the robustness of the topology obtained.

### 4.6. Gene Expression

Expression analysis of the carbonic anhydrases FRAAL1222, *can1* and FRAAL4889, *can2;* carbonate transporter FRAAL4724, *ctp* and two carbamoylphosphate synthase genes, FRAAL4883, (*cspA*) and FRAAL4884, (*cspB*), was carried out with cells grown on FBM+ medium [54] with 5 mM propionate and with 5 mM ammonium chloride and cells grown on FBM− medium without ammonium chloride and in 21 dpi nodules [21]. This was monitored by qPCR using the primers that are listed in Table 4 and following the procedure described previously [21].

### 4.7. Proteomics

Proteomic analysis of 5 mM propionate-fed and 5 mM fumarate-fed *Frankia* cells grown in BAP- was carried out as described before [60]. The mass spectrometry proteomics data have been deposited in the ProteomeXchange Consortium via the PRIDE partner repository with the dataset identifiers PXD018045 and 10.6019/PXD018045.

### 4.8. Metabolomic of Organic Acids

The organic acids in the nodules and roots were quantified using 22-day-old nodules grown as described in Alloisio et al. [31]. These were extracted from liquid nitrogen ground tissues. Sterile ultrapure water was added to achieve a concentration of 1 g of nodule per mL of water. Samples were centrifuged at 13,000 rpm in Eppendorf centrifuge for 10 min.

After extraction, the supernatants of the nodules or roots were diluted (1:20) in ultrapure water and filtrated on a 0.45 µm PVDF filter prior to being analysed by ionic chromatography and tandem mass spectrometry (IC-MS/MS). IC-MS/MS was used to verify the presence of organic acids already quantified by IC-conductivity and to identify other unknown organic acids.

The IC-MS/MS instrument involved was the ICS-5000^+^ Ion Chromatography System combined with a TSQ Fortis Triple Quadrupole Mass Spectrometer (all Thermo Scientific, Waltham, MA, USA). The monitoring software was Chromeleon 7.2.10. The IC system was equipped with an AS-AP auto-sampler, a Dual Pump analytical gradient system, a suppressor ADRS 600 (2 mm, Thermo Scientific) and a conductivity detector (P/N 061830). The IC separation was realized using an anion-exchange column IonPac AS11-HC-4 µm (2 × 250 mm^2^, Thermo Scientific, Illkirch, France) preceded by a guard column IonPac AG11-HC-4 µm (2 × 50 mm^2^, Thermo Scientific) and with the same parameters as the IC-conductivity analysis. Concerning the mass spectrometer, the heated electrospray ionization source was used in negative mode with the following parameters: negative ion spray voltage 3.0 kV, sheath gas 52 Arb, auxiliary gas 13 Arb, sweep gas 16 Arb, ion transfer tube temperature 275 °C and vaporizer temperature 375 °C. Acquisitions were realized in full scan mode or in Single Ion Monitoring mode with analytes detected after deprotonation in ionization source.

The flow chart of Materials and Methods is presented in Supplementary Figure S1.

## Figures and Tables

**Table 1:** List of genes conserved between *Frankia* strains of clusters 1, 2 and 3 but absent in cluster 4 genomes at a level of 50% Id. Bold letters indicate carbonic anhydrase.

Label	Begin	Gene	Product
FRAAL0011	12280	-	putative membrane protein
FRAAL0337	365258	*ddpC*	ABC transporter dipeptide permease, membrane component
FRAAL0338	366130	*ddpB*	ABC transporter oligopeptide permease, membrane component.
FRAAL0476	543271	*-*	Putative acetyltransferase
FRAAL0564	622164	*-*	Protein of unknown function
FRAAL0674	735035	*-*	Protein of unknown function
FRAAL0757	818794	*-*	Putative membrane protein
FRAAL0911	981382	*malE*	Malic enzyme
FRAAL1009	1080832	*-*	Protein of unknown function
FRAAL1027	1099037	*-*	6-aminodeoxyfutalosine deaminase
FRAAL1029	1101440	*-*	Protein of unknown function
FRAAL1057	1133454	*-*	Putative membrane protein
FRAAL1119	1186573	*-*	Putative PadR-like family Transcriptional regulator
FRAAL1194	1269992	*deoA*	Thymidine phosphorylase
**FRAAL1222**	**1300029**	***can***	**Carbonic anhydrase**
FRAAL1671	1792140	*-*	Putative oxidoreductase
FRAAL1675	1796237	*-*	Oxidoreductase involved in polyketide synthesis
FRAAL1914	2074787	*folC*	Folylpolyglutamate synthase, Dihydrofolate synthase
FRAAL1958	2125395	*-*	Putative Short-chain dehydrogenase/reductase SDR
FRAAL1997	2164212	*-*	Putative DNA-binding protein
FRAAL2052	2228153	*gtf*	UDP-Glycosyltransferase
FRAAL2055	2231919	*idhA*	Inositol 2-dehydrogenase
FRAAL2057	2235712	*rffE*	UDP-N-acetyl glucosamine-2-epimerase
FRAAL2100	2290841	*-*	Protein of unknown function
FRAAL2126	2322837	*trpE*	Anthranilate synthase
FRAAL2162	2363687	*-*	Conserved protein of unknown function
FRAAL2208	2420975	*lspA*	Lipoprotein signal peptidase
FRAAL2318	2525122	*-*	Protein of unknown function
FRAAL2432	2651458	*-*	Protein of unknown function
FRAAL2718	2967613	*-*	conserved hypothetical protein
FRAAL2942	3184379	*-*	conserved hypothetical protein
FRAAL3047	3321125	*-*	Protein of unknown function
FRAAL3189	3459207	*-*	Transcriptional regulator, lysR family
FRAAL3191	3462339	*-*	Monooxygenase FAD-binding
FRAAL3194	3471740	*fabA*	beta-ketoacyl synthase
FRAAL3195	3473917	*pksE*	Polyketide biosynthesis protein PksE
FRAAL3196	3475554	*accA*	Acetyl carboxylase
FRAAL3211	3491576	*-*	Putative phytoene dehydrogenase (Phytoene desaturase)
FRAAL3225	3507852	*pheA*	Prephenate dehydratase (PDT)
FRAAL3280	3568827	*-*	Putative NADPH-dependent FMN reductase domain
FRAAL3449	3738250	*-*	Putative fatty acid desaturase
FRAAL3451	3739662	*-*	Cyclopropane-fatty-acyl-phospholipid synthase
FRAAL3453	3742003	*-*	Putative peptide synthetase
FRAAL3533	3831802	*-*	Putative GntR-family transcriptional regulator
FRAAL3599	3896141	*-*	Protein of unknown function
FRAAL4108	4444365	*-*	Protein of unknown function
FRAAL4206	4571703	*-*	Putative cytochrome P450
FRAAL4337	4705479	*sodF*	Superoxide dismutase [Fe-Zn] 1 (FeSOD I)
FRAAL4345	4713659	*-*	Protein of unknown function
FRAAL4378	4754901	*-*	putative DNA helicase
FRAAL4436	4823328	*-*	Membrane-bound metal-dependent hydrolase
FRAAL4513	4899008	*-*	Lysophospholipase; GDSL domain
FRAAL4612	5006448	*-*	Putative membrane protein; putative GGDEF and EAL domains
FRAAL4744	5135553	*-*	Conserved hypothetical protein
**FRAAL4889**	**5291471**	***can***	**Carbonic anhydrase**
FRAAL4971	5397536	*-*	Trp biosynthesis associated, transmembrane protein, Oprn/Chp
FRAAL5040	5472936	*-*	Putative acetyltransferase protein
FRAAL5142	5574524	*cobS*	cobalamin 5′-phosphate synthase
FRAAL5202	5637735	*argG*	Argininosuccinate synthase (Citrulline-aspartate ligase)
FRAAL5248	5688660	*-*	Putative integral membrane peptidase
FRAAL5656	6113062	*-*	D-amino acid dehydrogenase small subunit
FRAAL5679	6141476	*mobA*	molybdopterin-guanine dinucleotide biosynthesis protein (partial)
FRAAL5702	6174425	*-*	Conserved hypothetical protein; putative signal peptide
FRAAL5757	6240667	*-*	Conserved hypothetical protein
FRAAL5768	6250966	*-*	ABC transporter transmembrane subunit
FRAAL5770	6252849	*-*	Aliphatic sulfonates family ABC transporter, periplsmic ligand-binding protein
FRAAL5844	6327476	*-*	Putative signal peptide
FRAAL6006	6512802	*-*	Putative permease
FRAAL6064	6579159	*-*	Putative PE-PGRS family protein PE_PGRS54 precursor
FRAAL6069	6583344	*-*	Putative integral membrane protein (partial match); putative coiled-coil domain
FRAAL6082	6599422	*-*	putative succinate-semialdehyde dehydrogenase I, NADP-dependent
FRAAL6136	6660036	*-*	putative pterin-4-alpha-carbinolamine dehydratase (PHS)
FRAAL6207	6732283	*-*	Conserved protein of unknown function, putative coiled-coil domain
FRAAL6220	6748530	*-*	Putative Prolyl aminopeptidase
FRAAL6231	6759567	*-*	Conserved hypothetical protein
FRAAL6294	6828148	*-*	Conserved protein of unknown function
FRAAL6420	6974297	*lysA*	Diaminopimelate decarboxylase protein
FRAAL6421	6976208	*rhbA*	Diaminobutyrate-2-oxoglutarate aminotransferase
FRAAL6422	6977644	*rhbB*	L-2,4-diaminobutyrate decarboxylase
FRAAL6424	6981251	*rhbE*	rhizobactin biosynthesis protein RhbE
FRAAL6425	6982828	*rhbF*	N4-acetyl-N4-hydroxy-1-aminopropane citrate synthase, siderophore
FRAAL6426	6985113	*glnA3*	Glutamine synthetase
FRAAL6442	7009938	*hesA2*	Protein hesA
FRAAL6448	7014231	*-*	Putative molybdenum binding protein
FRAAL6506	7073847	*mmpI*	cAMP-binding potassium voltage-gated channel protein
FRAAL6507	7075397	*cyc2*	Germacradienol/geosmin synthase
FRAAL6536	7112699	*-*	Conserved protein of unknown function
FRAAL6606	7192461	*-*	Hypothetical protein; putative signal peptide
FRAAL6623	7206003	*-*	Putative F420-dependent glucose-6-phosphate dehydrogenase domain
FRAAL6637	7231846	*-*	Conserved hypothetical protein; putative NERD domain
FRAAL6653	7250627	*-*	Secreted CAP protein
FRAAL6671	7268730	*panD*	Aspartate 1-decarboxylase
FRAAL6724	7333518	*purQ*	Phosphoribosylformylglycinamidine synthase I (FGAM synthase I)
FRAAL6730	7338262	*purL*	Phosphoribosylformylglycinamidine synthase II (FGAM synthase II)
FRAAL6738	7347692	*-*	Conserved hypothetical protein
FRAAL6800	7407340	*nifS*	Cysteine desulfurase (Nitrogenase metalloclusters biosynthesis protein)
FRAAL6801	7408818	*hesA*	Molybdenum cofactor biosynthesis protein hesA
FRAAL6802	7409711	*erpA*	Iron-sulfur cluster insertion protein ErpA
FRAAL6803	7410328	*nifB*	FeMo cofactor biosynthesis protein nifB
FRAAL6805	7412516	*nifW*	Nitrogenase stabilizing/protective protein nifW
FRAAL6807	7413131	*-*	Putative NifX-associated protein
FRAAL6808	7413571	*nifX*	NifX protein
FRAAL6810	7415792	*nifE*	Nitrogenase iron-molybdenum cofactor biosynthesis protein nifE
FRAAL6811	7417374	*nifK*	Nitrogenase molybdenum-iron protein beta chain
FRAAL6812	7419136	*nifD*	Nitrogenase molybdenum-iron protein alpha chain
FRAAL6813	7420646	*nifH*	Nitrogenase iron protein (Nitrogenase component II)
FRAAL6814	7421686	*nifV*	Nitrogenase-associated homocitrate synthase
FRAAL6857	7460193	*-*	Peptidoglycan-binding glycosyltransferase

**Table 2:** List of the 40 most overabundant proteins in fumarate-fed cells (section a) or in propionate-fed cells (section b).

Section a	40 Most Overabundant Proteins in Fumarate-Fed Cells Relative to Propionate-Fed Cells	
Gene	Functional description	Tfold
FRAAL4675	Acyl-CoA transferase	11.5
FRAAL2567	Protein of unknown function	8.5
FRAAL3063	Branched-chain amino acid ABC transporter	7.4
FRAAL1390	C4-dicarboxylate transporter	6.83
FRAAL3378	Protein of unknown function	6.8
FRAAL0382	CoA-transferase	6.35
FRAAL6022	Gamma-aminobutyraldehyde dehydrogenase	6
FRAAL4493	Alkyl hydroperoxide reductase AhpD	5.6
FRAAL3042	Short-chain dehydrogenase	5.23
FRAAL4031	Protein of unknown function	5
FRAAL2915	Dehydrogenase	4.5
FRAAL5988	Arginine ABC transporter ATP-binding protein	4.4
FRAAL6577	Potassium transporter	4.37
FRAAL0095	Large mechanosensitive ion channel protein MscL	4.2
FRAAL4431	Clp protease	4
FRAAL5296	Protein of unknown function	3.86
FRAAL4754	Protein of unknown function	3.83
FRAAL2473	Antibiotic biosynthesis monooxygenase	3.8
FRAAL0785	Membrane protein of unknown function	3.8
FRAAL4195	Protein of unknown function	3.8
FRAAL3527	Branched-chain amino acid ABC transporter	3.71
FRAAL2449	F420-dependent oxidoreductase	3.69
FRAAL4251	ABC transporter ATP-binding protein	3.6
FRAAL3695	Deaminase	3.6
FRAAL5155	Peptidase	3.55
FRAAL3391	Branched-chain amino acid ABC transporter	3.43
FRAAL3545	Protein of unknown function	3.4
FRAAL0756	C4-dicarboxylate ABC transporter	3.4
FRAAL0729	Branched-chain amino acid ABC transporter	3.4
FRAAL4252	Nitrate ABC transporter permease	3.4
FRAAL0091	Membrane protein of unknown function	3.4
FRAAL4026	Short-chain dehydrogenase	3.38
FRAAL6394	Molybdenum cofactor biosynthesis protein	3.33
FRAAL3575	Protein of unknown function	3.33
FRAAL4335	C4-dicarboxylate ABC transporter	3.3
FRAAL2821	Peptide-binding protein	3.27
FRAAL3848	Thiolase	3.2
FRAAL3365	Branched-chain amino acid ABC transporter	3.2
FRAAL5858	Amino acid-binding protein	3.17
FRAAL2285	Phosphohistidine phosphatase	3.14
FRAAL2855	Protein of unknown function	3.14
**Section b**	**40 most overabundant proteins in propionate- relative to fumarate-fed cells**	
Gene	Functional description	Tfold
FRAAL3447	Beta-ketoacyl synthase	10.8
FRAAL4683	Carbamoyl phosphate synthase large subunit	10.8
FRAAL6742	Protein of unknown function	8.2
FRAAL3139	Protein of unknown function	7.8
FRAAL6810	Nitrogenase iron-molybdenum cofactor biosynthesis NifE	7.6
FRAAL4690	Protein of unknown function	7.12
FRAAL4693	Inducer of phenazine A	6.67
FRAAL4681	Tryptophan synthase subunit beta	6.6
FRAAL5683	DNA-binding protein	6.1
FRAAL4934	Acetyl-CoA acetyltransferase	6.08
FRAAL4684	Carbamoyl phosphate synthase small subunit	5.8
FRAAL4687	L-amino acid ligase	5.61
FRAAL0025	Glycosyl transferase family 1	5.6
FRAAL6528	DNA repair protein RadA	5.2
FRAAL2491	Squalene-hopene cyclase	5.2
FRAAL5157	Lipoyl synthase	5.2
FRAAL4695	Oxidoreductase	5.16
FRAAL1433	4-hydroxy-3-methylbut-2-enyl diphosphate reductase	5.12
FRAAL4489	Carboxylate-amine ligase	5.08
FRAAL4685	Biotin carboxylase	4.8
FRAAL6803	Nitrogen fixation protein NifB	4.68
FRAAL0263	Protein of unknown function	4.67
FRAAL4691	Protein of unknown function	4.6
FRAAL4724	Putative carbonate transporter	4.6
FRAAL2577	Protein of unknown function	4.6
FRAAL6814	Homocitrate synthase	4.33
FRAAL6720	Preprotein translocase SecA	4.33
FRAAL0984	Porphobilinogen deaminase	4.33
FRAAL5851	Acetolactate synthase	4.25
FRAAL1432	Squalene-hopene cyclase	4.23
FRAAL5482	Acetyl-CoA acetyltransferase	4.22
FRAAL1003	Cytochrome C biogenesis protein ResB	4.2
FRAAL6672	L-aspartate oxidase	4.2
FRAAL4095	FMN-dependent alkanal monooxygenase	4.2
FRAAL3600	Protein of unknown function	4.18
FRAAL5337	(2Fe-2S)-binding protein	4.18
FRAAL1423	Protein of unknown function	4.17
FRAAL0262	Helicase	4.1
FRAAL5952	Diaminopimelate decarboxylase	4
FRAAL6862	HDIG domain-containing protein	4
FRAAL3851	Serine/threonine protein kinase	4

**Table 3:** Abundance of organic acids in *A. glutinosa* nodules (N1 and N2) and roots (R1 and R2).

Compound	Formula	R1	R2	N1	N2	Ratio (N1 + N2)/(R1 + R2)
Formic acid	HCOOH	235,661	76,769	162,492	117,920	0.90
Acetic acid	CH_3_COOH	4997	605	5543	3227	1.57
Pyruvic acid	CH_3_COCOOH	70,735	16,847	78,529	87,003	1.89
Propionic acid	CH_3_CH_2_COOH	1511	920	948	808	0.72
Malonic acid	CH_2_(COOH)_2_	418,776	125,492	441,849	330,058	1.42
Malic acid	C_4_H_6_O_5_	6,842,086	5,418,694	7,713,529	7,219,222	1.22
Succinic acid	(CH_2_)_2_(COOH)_2_	18,095	18,081	20,557	21,713	1.17
Oxoglutaric acid	C_3_OH_4_(COOH)_2_	165,722	32,647	230,854	189,843	2.12
Fumaric acid	HOOC-CH=CH-COOH	7867	2193	11,919	8994	2.08
Citric acid	HOC(COOH)(CH_2_COOH)_2_	11,042,145	9,255,806	14,287,742	11,434,886	1.27
Oxalic acid	HOOC-COOH	667,609	738,765	811,020	672,537	1.05

**Table 4:** List of the primers used for monitoring the expression level of genes in symbiosis and in fumarate-fed and propionate-fed cells.

Oligo	Sequence
FRAAL1222q7	AGATCATCCTCCTGCACCACA
FRAAL1222q8	GAAGCCGTCGTCGGTGAT
FRAAL4889q5	GCTGGGAACCCGAGAGATCA
FRAAL4889q6	GAACGCCTCGTCGGTGAAAG
FRAAL4724q1	TCCTCGGTCAGATCCACGTC
FRAAL4724q2	GTATCGCCCGGATGTTGTCC
FRAAL4684q3	TGCCTCGGTAACCAACTCCT
FRAAL4684q4	TCCTGGACCGGCTGATTGAT
FRAAL4683q5	ACTGGTGACTCCATCACGGT
FRAAL4683q6	CGATGTCGCGAAGGATCTGG
16sRNA_f	GAGTAACACGTGGGCAACCT
16sRNA_r	ATCCCGAGCCGATAAATCTT

## Data Availability

The original contributions generated for this study are included in the article; further inquiries can be directed to the corresponding authors.

## Supplementary Materials

The following supporting information can be downloaded at: https://www.mdpi.com/article/10.3390/ijms24119162/s1.
