# Supplementary material for: Frankia alni Carbonic Anhydrase Regulates Cytoplasmic pH of Nitrogen-Fixing Vesicles
Source: Int J Mol Sci. 2023 May 23;24(11):9162. doi: 10.3390/ijms24119162 (PMC10252897; doi:10.3390/ijms24119162)

Supplementary Table S1. List of strains used to search for genes specific to symbiotic *Frankia* genomes.

Symbiotic strains

Cluster 1 strains. Able to form effective nodules on *Alnus* and *Myricaceae*. Most strains can grow in pure culture on TCA intermediates except a few lineages called Sp+ not considered here. They have *nif* genes and can fix N2 in pure culture.

ACN14a (*Frankia alni,* isolated from *Alnus*, infective on *Alnus*)
QA3 (undescribed species, isolated from *Alnus*, infective on *Alnus*)
CcI3 (*Frankia casuarinae,* isolated from *Casuarina*, infective on *Casuarina*)

Cluster 2 strains. Able to form effective nodules on *Datisca, Coriaria* and *Ceanothus*. Most strains cannot be grown in pure culture except one BMG5.1 that can fix N2 in pure culture. They have *nif* genes and fix N2 in nodules.

BMG5.1 (*Frankia coriariae,* isolated from *Coriaria*, infective on *Coriaria*)
Dg1 (*Candidatus* Frankia datiscae*,* not isolated, infective on *Datisca*)

Cluster 3 strains. Able to form effective nodules on *Elaeagnaceae* and *Gymnostoma*. Most strains can grow in pure culture on TCA intermediates and on hexoses. They have *nif* genes and can fix N2 in pure culture.

EAN1pec (*Frankia soli,* isolated from *Elaeagnus*, infective on *Elaeagnus*)
EUN1f (undescribed species, isolated from *Elaeagnus*, infective on *Elaeagnus*)

Non-Symbiotic strains

Cluster 4 strains. Can be grown in pure culture but are not symbiotic. Some (CN3 and DC12) although isolated from nodules cannot reinfect their host and cannot fix N2 in pure culture. Others (AgB1.8 and EuI1c) can reinfect their host but cannot fix N2 either in nodules nor in pure culture. They do not have *nif* genes.

CN3 (*Frankia saprophytica*, isolated from *Coriaria*, non-infective). Used to subtract.
DC12 (undescribed species, isolated from *Datisca*, non-infective). Used to subtract.
AgB1.8 (isolated from *Alnus*, infective on *Alnus*, non-effective). Not used to subtract.
EuI1c (isolated from *Elaeagnus*, infective on *Elaeagnus*, non-effective). Not used to subtract.

Supplementary Figure S1. A flow chart of research and methods used in this work from comparative genomics to experimental studies.

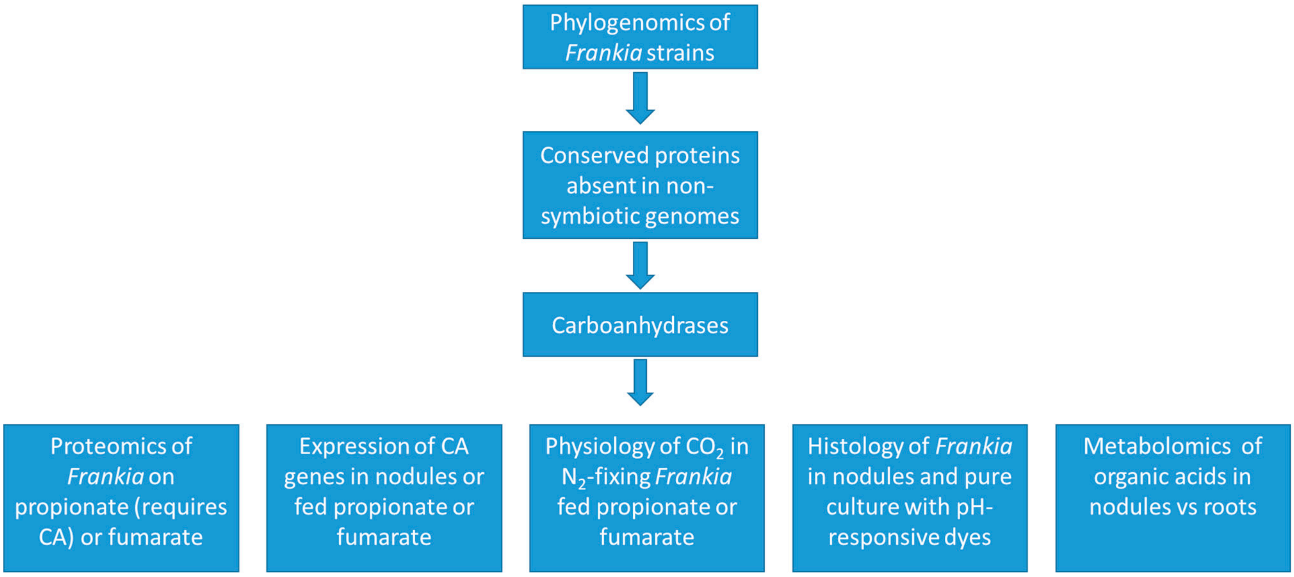

Supplement: Supplementary file 1 [file ijms-24-09162-s001.zip › ijms-2379398-supplementary.pdf]
